# IME_Rho_tet, a novel family of putative integrative mobilizable elements spreading tetracycline resistance genes tet(W) and tet(32) among human and animal gut bacteria

**DOI:** 10.1099/mgen.0.001640

**Published:** 2026-02-13

**Authors:** Ouleye Sidibé, Benoit Doublet, Sébastien Olivier Leclercq

**Affiliations:** 1 UMR ISP, INRAE, Université François Rabelais de Tours, F-37380 Nouzilly, France

**Keywords:** gut microbiota, mobile genetic element, *tet*(W), *tet*(32)

## Abstract

Antimicrobial resistance remains a major global health concern in human and animal medicine. Tetracycline resistance genes of the ribosomal protection protein (RPP) family, especially *tet*(W), are among the most abundant resistance genes in livestock animals and human gut microbiota. However, genetic determinants responsible for the spread of *tet*(W) are not yet fully described. Here, all genomes of the phyla *Bacillota* and *Actinomycetota* available in RefSeq were screened for Tet(W)-related proteins as well as signature proteins of a new putative integrative mobilizable element (IME) recently described to carry the *tet*(W) gene. This element, hereafter named IME_Rho_tet, showed an extensive diversity, both for its relaxase and serine recombinase signature proteins and for the encoded tetracycline resistance protein. A total of 504 complete and 1,090 partial IME_Rho_tet elements were detected, of which 68% encoded Tet(W). The remaining IME_Rho_tet encoded other RPPs, including Tet(32), Tet(O) and the mosaic Tet(O/32/O), as well as a few novel RPP and variants. Overall, 71% of all *tet*(W) detected in bacteria isolated from human or animal gut were linked to IME_Rho_tet, as well as 87% of all *tet*(32). IME_Rho_tet elements showed an extensive bacterial host range, as complete elements were detected in more than 60 *Bacillota* genera, while partial elements were also detected in 20 *Actinomycetota* genera. Finally, eight of the ten mobile elements reported in the literature as carrying *tet*(W) or *tet*(32) included complete or partial IME_Rho_tet. These findings highlight the IME_Rho_tet family as a key genetic player in the dissemination of *tet*(W) and *tet*(32) among gut-associated bacteria of humans and food-producing animals.

**Impact Statement**

Mobile genetic elements are main drivers of horizontal transfer of antibiotic resistance genes (ARGs) between bacteria in rich microbial ecosystems such as gut microbiota. While antimicrobial resistance dissemination is well described for medically important resistances of clinically relevant pathogens, there is growing attention to the spread of ARGs within commensal gut bacterial communities. Tetracycline resistance genes are known to be some of the most abundant resistance genes in livestock animals and human gut microbiota. Using the wealth of bacterial genomes available in the RefSeq database, we identified a novel family of highly diverse integrative mobilizable elements that are responsible for the spread of *tet*(W) and *tet*(32) and a few other related resistance genes among gut-associated bacteria of humans and food-producing animals. These results provide for the first time an explanation for the wide distribution of *tet*(W) and *tet*(32) tetracycline resistance genes in *Bacillota* bacterial species associated with human and animal gut microbiota.

## Data Summary

Bioinformatics scripts and pipelines used to perform analyses are available on GitHub at https://github.com/OuleyeSidibe/IME_Rho_tet_Snakemake_Pipeline. All analyses were carried out on publicly available genomic sequences. Accession numbers and a summary of all RPP-encoding genes identified within complete, partial or outside of IME_Rho_tet are provided in Table S1 (Table S1).

## Introduction

Pathogenic bacteria resistant to multiple antibiotics are one of the major public health concerns of the 21st century, with 1.14 million deaths attributable to antibiotic resistance worldwide in 2021 [1]. Most critical emergences of resistance are linked to the acquisition of mobile genetic elements (MGEs) carrying antibiotic resistance genes (ARGs), which spread in bacterial populations exposed to antibiotics [2]. It is now widely recognized that pathogens acquire these MGEs from non-pathogenic bacteria from the environment or from human and animal-associated microbiota [2]. Among them, the gut microbiota of humans and animals have been considered as a major reservoir of ARGs due to their richness and diversity of bacterial species, their tight connexion with several alarming nosocomial germs such as pathogenic *Escherichia coli*, *Klebsiella pneumoniae* and *Enterococcus* spp. and their recurrent exposure to numerous antibiotics, especially for industrially farmed animals extensively treated with antibiotics for decades [3]. The taxonomic composition of gut microbiota of humans and main livestock animals is similar, with a majority of taxa from the *Bacillota* (formerly *Firmicutes*) and *Bacteroidota* (formerly *Bacteroidetes*) phyla, plus some *Pseudomonadota* (formerly *Proteobacteria*) and *Actinomycetota* (formerly *Actinobacteria*) at low abundance [4,7]. Until recently, most anaerobic members of the gut microbiota remained uncultured, and our knowledge of ARGs present in commensals was limited to those circulating in a few cultivable bacteria such as *E. coli*, *Enterococcus faecalis* and some *Bacteroides* spp. [8]. The full extent of ARG load in gut microbiota was revealed with the first population-scale metagenomics analyses, providing a general picture of ARGs found in the gut of healthy humans and farm animals [9,12]. Besides the high diversity of detected ARGs, these studies pointed out the extreme prevalence and abundance of a few genes, especially those of the ribosomal protection protein (RPP) class of tetracycline resistance genes. For instance, *tet*(O), *tet*(Q), *tet*(W) and *tet*(32) were detected in all screened gut metagenomes from 162 humans, 181 pigs and 178 chickens originating from various countries [9,11]. Among them, *tet*(W) is frequently detected as the most abundant or most disseminated ARG in human [12,13], chicken [14,15], pig [11], cattle [16] and horse [17] gut microbiota. This gene was first described in 1999 in *Butyrivibrio fibrisolvens* 1.230 isolated from cow rumen and was shown to be also present in *Selenomonas ruminantium* isolated from sheep rumen and *Mitsuokella multiacidus* isolated from pig gut [18]. It was then reported in various gut and oral microbial species from animals and humans [19,21] and soon became the RPP gene with the second largest known host spectrum after *tet*(M), spanning *Bacillota* and *Actinomycetota* phyla [14,22]. Despite its widespread presence in commensal bacterial species, the genetic determinants contributing to *tet*(W) dissemination in gut microbiota are still unclear. In *B. fibrisolvens*, it was shown to be transferable between strains through a 40–50 Kbp conjugative genomic island called Tn*B1230* [18], for which only the 12 Kb region surrounding *tet*(W) was sequenced [23]. However, Tn*B1230* has never been detected in any other bacterial species. In *Trueperella pyogenes* (formerly *Arcanobacterium pyogenes*), an *Actinomycetota* commensal of livestock animals, *tet*(W) is carried by ATE-1, an integrative mobilizable element (IME) which has been described to horizontally transfer at low frequency to other *T. pyogenes* and exists in several structural variants [24,25]. Other *tet*(W) variants were also described in two other putative small transposons in *T. pyogenes*, ATE-2 and ATE-3, but no transferability could be detected [26]. With the advent of bacterial genome sequencing, new *tet*(W)-carrying MGEs were reported, including ΦssUD.1, ICE_lysS and Tn*5252* in *Streptococcus suis* [27,29], the plasmid pJA144188 in *Corynebacterium striatum* [30], the putative integrative and conjugative element (ICE) ICE_RbtetW_07 in *Blautia schinkii* DSM 10518 [16] and the putative integrative and mobilizable element (IME) IME_RhoA2-183_maff2-1 in *Roseburia hominis* A2-183 [31]. This latter element is of particular interest because it is identical in structure to a putative *tet*(W)-carrying MGE described in several genomes of human and animal gut bacterial species [32] and may therefore have a critical role in the observed prevalence of *tet*(W) in gut microbiota.

In the present study, we investigated the diversity and distribution of IME_RhoA2-183_maff2-1, hereafter renamed IME_Rho_tet, in all RefSeq genomes of commensal *Bacillota* and *Actinomycetota*. Overall, 70% of all *tet*(W) detected in gut-associated bacterial genomes are carried by or originate from IME_Rho_tet, as well as 87% of all *tet*(32). We also show that most *tet*(W) previously reported on other MGEs, such as ICE_RbtetW_07 and Tn*B1230*, are part of or originate from IME_Rho_tet.

## Methods

### Genomic data collection

All species belonging to phyla *Bacillota* and *Actinomycetota* were identified from the National Center for Biotechnology Information (NCBI) taxonomic file downloaded in June 2024 [33]. For 14 bacterial species (*Bacillus cereus*, *Clostridioides difficile*, *Enterococcus faecalis*, *E. faecium*, *Lactiplantibacillus plantarum*, *Listeria monocytogenes*, *Mycobacterium tuberculosis*, *Mycobacteroides abscessus*, *Staphylococcus aureus*, *Staphylococcus epidermidis*, *Streptococcus agalactiae*, *Streptococcus pyogenes*, *Streptococcus suis* and *Streptococcus pneumoniae*), all available RefSeq assemblies were retrieved from the NCBI database in November 2025. All RefSeq assemblies for the remaining species were retrieved from the INRAE bioinformatics platform Migale in June 2024[34].

### *In silico* search of IME_Rho_tet

Complete IME_Rho_tet elements were identified by performing a blastp search (version 2.13.1) [35] against annotated proteins of each assembly, using the RPP (WP_014078531), relaxase (WP_014078536) and recombinase (WP_014078528) as signature proteins from the canonical sequence IME_RhoA2_183_maff2-1 of *R. hominis* (NC_015977) [31]. blastp hits were filtered using minimum thresholds of 50% sequence identity and 90% coverage. All genomes containing at least one relaxase that passed the filter thresholds were searched for IME_Rho_tet elements using a co-localization method with the relaxase as the anchor point. This method consisted of determining whether the signature proteins were physically located in the same genomic region (Fig. S1, available in the online Supplementary Material). For each complete genome or contig containing a relaxase, we searched for the presence of a recombinase within ten coding DNA sequences (CDS) downstream using CDS numbering provided in the protein annotation file. If a recombinase was detected, the search for an RPP was performed within ten CDS upstream of the relaxase. If an RPP was found, the sequence was defined as a complete IME_Rho_tet. Contigs that did not extend sufficiently to cover both upstream and downstream intervals of ten CDS around the relaxase were excluded from the analysis. All other RPPs detected on contigs or complete genomes were categorized as not on complete IME_Rho_tet.

### Protein clustering

Three protein databases corresponding to each of the signature proteins were constructed and subsequently clustered using CD-HIT v.4.8.1 [36] with a sequence identity threshold of 90%. The 56 protein sequences of the RPP class referenced in the ResFinder database v.4.0 [37], including 25 mosaic-type variants, were added to the RPP database prior to clustering to serve as references for the clusters to which they belong. For RPP clusters without such reference assignment, a blastp search against ResFinder was performed to determine whether the cluster representative corresponded to a new variant of a previously described protein or to a novel protein, based on the tetracycline resistance gene nomenclature guidelines [38]. Novel RPP variants were named with the suffix ‘newVar’ after the most similar protein (≥80% identity), while novel proteins were named ‘RPP_new’ followed by a number. Protein clusters of relaxases and recombinases are referred to as REL and REC, respectively.

### Boundaries and integration site characterization

Regions located upstream of the RPP gene and downstream of the recombinase gene of IME_Rho_tet described in the literature in *R. hominis* A2-183 [31], in ICE_RbtetW_07 of *Blautia schinkii* DSM 10518 [16] and in the chromosome of *Clostridium* sp. SY8519 [32] was aligned with clustalW [39] to determine potential boundaries. Terminal inverted repeats (TIRs) were identified at homology breaks and were used as input motifs to scan assemblies of IME_Rho_tet-carrying and RPP-carrying genomes with the FIMO program from the MEME suite (version 5.5.7) [40]. Scans were performed with an e-value threshold of 10^−9^, over regions extending 6 kb upstream and downstream of the RPP and recombinase genes, respectively. For all genomes with a TIR identified at both 5′- and 3′-ends, a 300 bp region corresponding to the integration site was reconstructed, spanning 150 bp upstream and 150 bp downstream of the TIRs. After dereplication with the program CD-HIT (minimum similarity threshold: 90%), a blastn search (version 2.16.0) against the NCBI nucleotide collection (nr) database was performed to identify homologous sequences. For each reconstructed region, the best hit was selected, and results were filtered to ensure a minimum of 97% coverage and 90% identity. Annotations within homologous sequences were then extracted from RefSeq, or from GenBank for sequences not annotated in RefSeq, in order to identify putative CDS overlapping the integration site (at position 150 bp, with a tolerance of ±10 bp). Proteins encoded by identified CDS were grouped into families based on their predicted function (CDS product feature). All proteins sharing the same functional designation were considered to belong to the same protein family.

### Sources analysis

Isolation source of each bacterium in the original dataset was identified from the ‘isolation_source’ tag provided in their RefSeq genome annotation. Bacteria were categorized as originating from gut if the tag included at least one of these keywords (case insensitive and with * as wildcards): gut, intestin*, digesti*, faeces, feaces, faecal, feace, stool, caecum, caeca, ceacal, ceca, cecum, duodenum, jejunum, rumen, colon, ileum, ileal, rectum, rectal, cloaca, chyme, manure. If the tag was empty, filled with ‘not provided’, ‘not available’, ‘biological product’, ‘glycerol stored’, ‘rich broth’, ‘clinical isolate’, ‘anaerobe media’ or including only a host name, the source was categorized as unknown. Otherwise, the source was categorized as non-gut. Hosts of gut-associated bacteria were identified from the ‘host’ tag of their RefSeq genome annotation. Keywords to set the category to ‘human’ were as follows: human, homo sapiens, hoimo sapiens, man, woman, adult, infant, baby, people. Keywords to set the category to ‘cattle’ were as follows: bovin*, cow, cattle, bos taurus, heifer, calf, beef, steer, charolais, holstein, limousine, angus, hereford. Keywords to set the category to ‘chicken’ were as follows: chick*, hen, broiler, gallus gallus, fowl, cockerel, poultry, rooster. Keywords to set the category to ‘pig’ were as follows: pig, swine, sus scrofa, suidae, hog, sow, pork. Keywords to set the category to ‘other domestic’ were as follows: duck, anas, turkey, meleagris, goose, anser, mouse, mus musculus, sheep, lamb, ovis, goat, capra, rat, rattus, rabbit, oryctolagus, bunny, hamster, mesocricetus, guinea pig, cavy, cavia, quail, mare, horse, pony, donkey, mule, equine, equus, cat, felis, dog, canis lupus familiaris, alpaca, vicugna pacos. Host was categorized as ‘unknown’ if not provided and ‘other’ otherwise. Bacterial genomes with an unknown isolation source or host were not considered in further analyses.

### Statistical tests

Independence between source distributions was calculated using Chi-Square tests with the Egon Pearson correction, allowing a better handling of small expected values [41]. Influence of each source on the distribution was estimated using the adjusted standardized residuals from the Egon Pearson correction test, and their statistical significance was adjusted for multiple testing (Hommel correction). All tests and *P* values were calculated using the R function epcs.test proposed by Campbell [42].

## Results

### Description of IME_Rho_tet

**Fig. 1.**
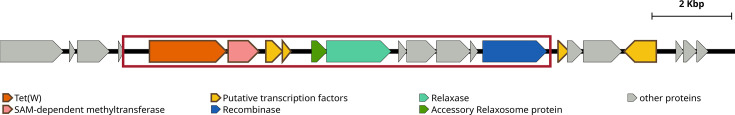
Canonical sequence defined in FirmiData for IME_Rho_tet in *R. hominis* A2-183 (Accession number NC_015977). The annotated IME_Rho_tet element is boxed in red. ORFs are coloured according to the protein they encode. Picture created using Genofig v.1.1 [57].

The canonical sequence defined in FirmiData for IME_Rho_tet in *R. hominis* A2-183 consists of 10,695 bp, including 11 open reading frames (ORFs) all in the same orientation and encoding typical IME-related modules such as a relaxase, the relaxosome accessory factor *mobC* and a serine recombinase [43]. It also encodes a class I SAM-dependent methyltransferase, two putative transcription factors and four proteins of unclear function in addition to the tetracycline resistance gene *tet*(W) (Fig. 1). Comparison with other copies of the element already reported in other gut bacterial species [32] suggests that the structure of IME_Rho_tet is well conserved, with only an additional putative endonuclease in some genomes (Fig. S2). On the other hand, a high degree of nucleotide divergence could be observed between copies for the relaxase and recombinase encoding genes, ranging from 3% to up to 20% depending on the gene (Fig. S2).

### Diversity of IME_Rho_tet

**Fig. 2.**
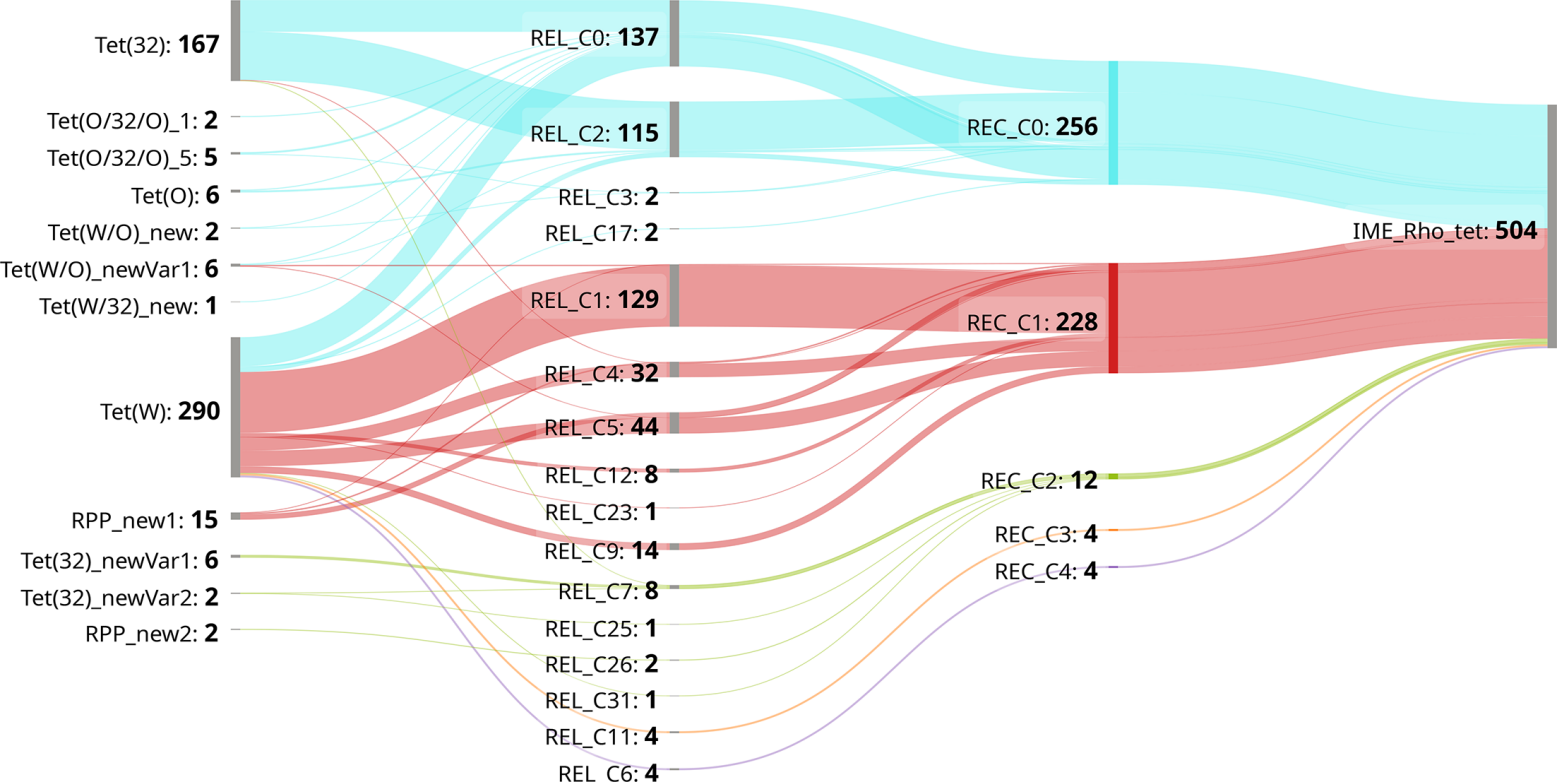
Associations among RPP, relaxase and recombinase clusters among the 504 identified IME_Rho_tet elements. Numerical values represent the number of IME_Rho_tet copies carrying each corresponding cluster. Links are coloured according to the recombinase cluster. Diagram created using SankeyMATIC [58].

According to the structure of IME_Rho_tet defined above, the relaxase, the recombinase and Tet(W) were selected as signature proteins to identify complete elements in 92,326 *Bacillota* and 30,457 *Actinomycetota* RefSeq genomes, using strict genomic co-localization and relaxed homology criteria (see Methods for details). This screening resulted in 504 detections in 117 genera. An extensive diversity was observed at the resistance gene locus, ranging from 67 to 99% amino acid similarity compared to the Tet(W) reference sequence (Table S2). Tetracycline RPP sequences were grouped in 12 clusters after clustering at 90% protein similarity and extracting mosaic genes (Table 1). The most abundant cluster corresponded to Tet(W) with 57.5% of the sequences, but to our surprise, the second most abundant cluster (33.1% of the sequences) corresponded to the protein Tet(32). Other clusters were very rare and included the known RPPs Tet(O) and Tet(O/32/O), two new distant variants of Tet(32), as well as two new putative RPPs and three new mosaics. IME_Rho_tet relaxases showed an even greater diversity with 16 identified clusters ranging from 63 to 100% protein similarity with the relaxase of IME_Rho_tet from *R. hominis* A2-183 (Table S3). Three clusters were found in more than 10% of the identified IME_Rho_tet, with the cluster REL_C0, containing the reference sequence, being the most represented, with 27.2% of all the relaxases. Finally, IME_Rho_tet recombinases were much more conserved, with sequences ranging from 85 to 100% protein similarity with the canonical sequence found in *R. hominis* A2-183, and were distributed in only five clusters (Table S4). Two clusters, including those of the reference sequence REC_C0, were almost equally represented and included 96% of the sequences. In total, 33 combinations of RPP–relaxase–recombinase clusters could be identified, but only 6 of them were dominant in our dataset, making up 81% of the detected IME_Rho_tet (Fig. 2). These combinations involved either REC_C0: Tet(32)-REL_C2-REC_C0 (100 copies), Tet(W)-REL_C0-REC_C0 (60 copies) and Tet(32)-REL_C0-REC_C0 (65 copies) or REC_C1: Tet(W)-REL_C1-REC_C1 (126 copies), Tet(W)-REL_C5-REC_C1 (32 copies) and Tet(W)-REL_C4-REC_C1 (28 copies). This indicates that the dissemination of resistance genes *tet*(W) and *tet*(32) is mostly driven by a few of the existing IME_Rho_tet variants.

**Table 1.** RPP clusters associated with IME_Rho_tet and their distribution within complete IME_Rho_tet, partial IME_Rho_tet or in other genetic contexts

RPP cluster	Complete IME_Rho_tet		Partial IME_Rho_tet		Other contexts		Total in IME_Rho_tet
	No.	(%)	No.	(%)	No.	(%)	(%)
Tet(W)	290	57.5	792	72.7	501	16.6	68.4
Tet(32)	167	33.1	38	3.5	29	1.0	87.6
Tet(O)	6	1.2	223	20.5	2,457	81.6	8.5
Tet(O/32/O)_1	2	0.4	3	0.3	1	0.03	83.3
Tet(O/32/O)_5	5	1.0	29	0.27	22	0.7	60.7
Tet(W/O)_new	2	0.4	–	–	–	–	100
Tet(W/O)_newVar1	6	1.2	–	–	–	–	100
Tet(W/32)_new	1	0.2	–	–	–	–	100
Tet(32)_newVar1	6	1.2	2	0.2	1	0.03	88.9
Tet(32)_newVar2	2	0.4	–	–	–	–	100
RPP_new1	15	3.0	3	0.3	–	–	100
RPP_new2	2	0.4	–	–	–	–	100
**Total**	**504**		**1,090**		**3,011**		

### Boundaries of IME_Rho_tet

**Fig. 3.**
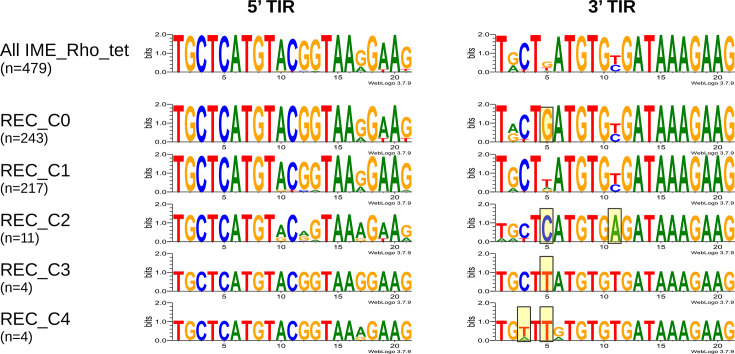
Sequence conservation of 5′ and 3′ TIRs for IME_Rho_tet with both TIRs detected, globally and separated by recombinase cluster. Both TIRs are represented 5′ to 3′. Boxed positions in 3′ TIRs represent recombinase-specific nucleotides. Picture created using WebLogo 3.7.9 [59].

Upstream and downstream regions of RPP- and recombinase-encoding genes of a representative subset of IME_Rho_tet were aligned to infer the precise boundaries of IME_Rho_tet elements. We identified a conserved 21 bp long motif found in inverted orientation between upstream and downstream regions, consistent with TIRs typical of recombination sites of serine recombinases [44]. A systematic search of the motif was then performed in all assemblies carrying IME_Rho_tet, which resulted in the detection of upstream and downstream TIRs for 479 (95%) out of the 504 IME_Rho_tet elements, while only upstream or downstream TIRs were detected for 15 and 8 elements, respectively. Overall, 3′ TIRs were less conserved than 5′ TIRs, especially at the second, fifth and eleventh positions (Fig. 3). Interestingly, the fifth position of the 3′ TIR is tightly linked to the recombinase cluster, with clusters C0, C2 and C3/C4 harbouring specifically a ‘G’, a ‘C’ and a ‘T’, respectively, suggesting a co-evolution between the recombinase and its cognate recombination site (Fig. 3). The distance between the 5′ boundary and the RPP-encoding gene varied between 391 and 3,109 bp (Fig. S3), with a distance of ~656 bp for 80% of IME_Rho_tet carrying *tet*(W). Most IME_Rho_tet carrying *tet*(O) and related mosaic genes showed a peculiar organization, with the RPP in reverse orientation and followed by *tet*(40) encoding a tetracycline efflux pump (Fig. S3). The 3′ region of IME_Rho_tet was much more conserved, with all 3′ boundaries lying 81–90 bp downstream of the recombinase stop codon.

### Identification of RPPs located on partial IME_Rho_tet or in other contexts

To understand the real contribution of IME_Rho_tet in RPP dissemination, the original RefSeq dataset was screened for instances of RPPs not located on complete IME_Rho_tet. A total of 1,293 additional Tet(W) copies were detected, as well as 67 Tet(32) and 2,680 Tet(O) additional copies (Table 1). Other RPPs were almost not found except Tet(O/32/O)_5 with 51 additional copies. A careful analysis of the 6 Kbp flanking the corresponding 4,101 RPP genes revealed that 1,090 (27%) could be considered as part of a partial IME_Rho_tet, as they were linked to one or both IME_Rho_tet TIRs (Table 1). Overall, 68.4, 87.6 and 60.7–100% of *tet*(W), *tet*(32) and low abundance RPPs were carried by complete or partial IME_Rho_tet, respectively, indicating that this element is their primary dissemination vector (Table 1). By contrast, only 8.5% of the detected Tet(O) were associated with IME_Rho_tet, consistent with its known localization on other mobile elements [28].

### Contribution of complete and partial IME_Rho_tet to the prevalence of RPPs in gut-associated bacterial genomes

**Fig. 4.**
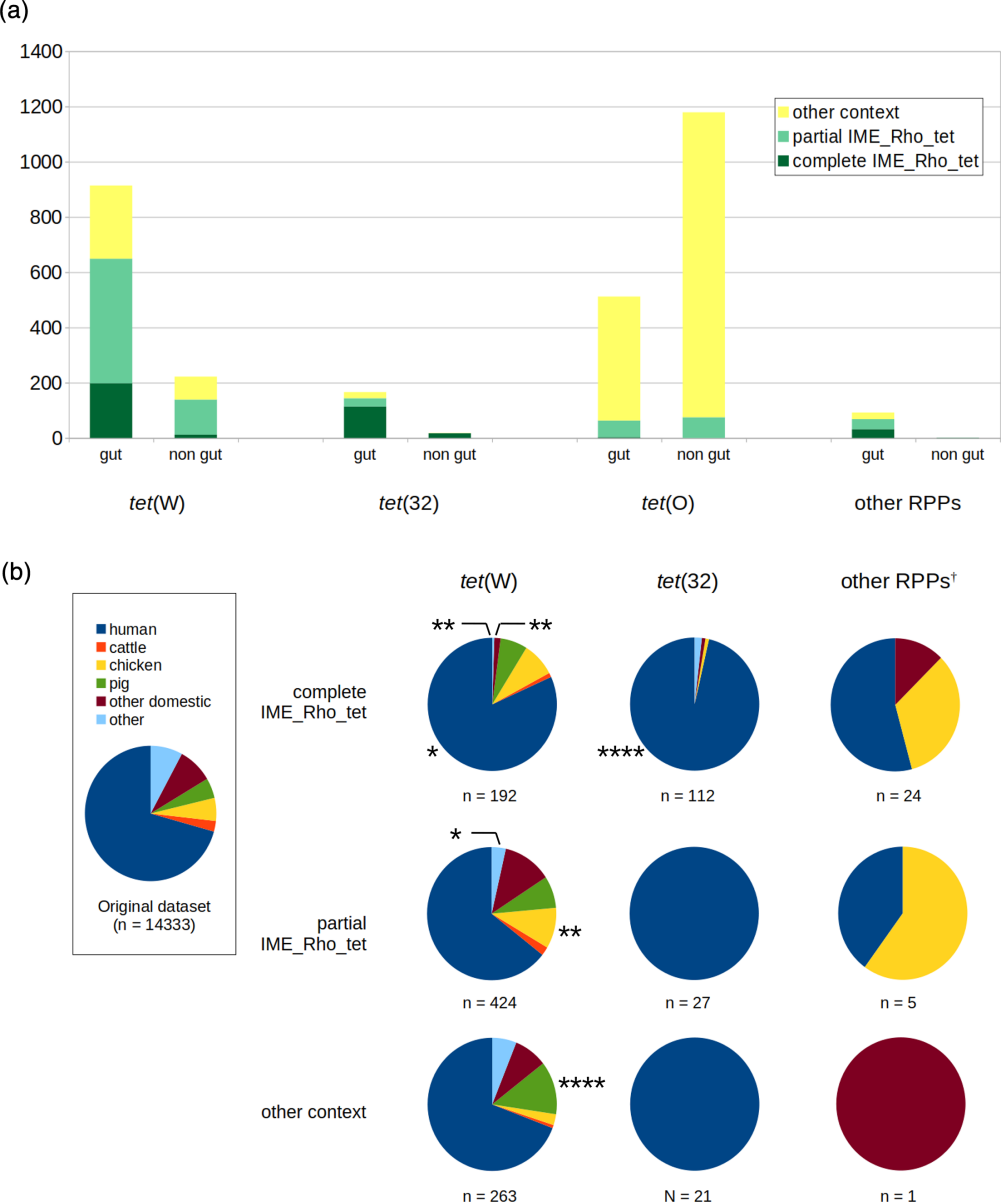
Source of bacteria carrying RPP genes. (a) Gut or non-gut origin of bacteria carrying *tet*(W), *tet*(32), *tet*(O) or other RPP genes. (b) Hosts from which gut bacteria were isolated. Human, cattle, chicken and pigs are represented independently, while other livestock animals and companion animals are grouped in ‘other domestic’. The group ‘other’ represents wild animals, including insects. †: *tet*(O) and *tet*(O/32/O) mosaic genes were not included in the ‘other RPPs’ group. Significant deviations from the original dataset host distribution were calculated on adjusted standardized residuals from an Egon Pearson test with Hommel correction for multiple tests. *: *P*<0.05; **: *P*<0.01; ****: *P*<10^−5^.

Since our original dataset included all *Bacillota* and *Actinomycetota* RefSeq genomes without ecological niche preselection, we ought to understand whether the 4,605 detected RPPs were preferentially found in gut-associated bacteria or not. An isolation source could be assigned to 68% of them, among which 55% were found in gut-associated bacterial genomes. This proportion was significantly higher than the 19% RefSeq genomes (15,913 out of 84,446, Eagon Pearson test *P*=0) originating from gut microbiota in the original dataset, indicating that, in general, RPPs analysed here were enriched in bacteria associated with the gut. However, this enrichment in gut bacteria was not homogeneous among RPPs, ranging from 30% (512/1,691) for Tet(O) up to 80% (914/1,136) for Tet(W), 90% (166/184) for Tet(32) and even 99% (92/93) for other RPPs (Fig. 4a). IME_Rho_tet contributed significantly more to Tet(W) prevalence in gut than in non-gut bacteria (71% vs. 63%, *P*=0.015), a trend also observed for Tet(O) (12% vs. 6%, *P*<10^−4^) (Fig. 4a). The contribution of IME_Rho_tet to Tet(32) prevalence was extremely high in both gut and non-gut bacteria, with no statistical difference (87% vs. 94%, *P*=0.35). These observations indicate that IME_Rho_tet contributed actively to the over-representation of the studied RPPs in gut-associated bacteria.

The 1,069 gut RPP-carrying bacterial genomes [excluding Tet(O) and related mosaic Tet(O/32/O) clusters] for which the host was known were then compared to the 14,333 gut bacterial genomes from the original dataset with an identified host. Hosts of gut bacteria bearing complete *tet*(W)-carrying IME_Rho_tet significantly differed from those of the original dataset (Eagon Pearson test *P*<10^−7^). Specifically, human origin was slightly over-represented, while animal origins other than cattle, pigs and chickens were highly under-represented (Fig. 4b). *tet*(W) genes located on partial IME_Rho_tet were significantly over-represented in bacteria of chicken gut origin compared to the original dataset (10% vs. 5.5%, Eagon Pearson test adjusted residuals *P*<0.01), to the extent of the association with bacteria of human gut origin. Finally, *tet*(W) genes in other genetic contexts were significantly more frequently found in gut bacteria of pig origin only compared to the original dataset (13% vs. 4.8%, *P*<10^−5^). *tet*(32) genes were carried essentially by gut bacteria of human origin, whether being associated with IME_Rho_tet or not (Fig. 4b), leading to a strong statistical difference compared to the original dataset when calculation was possible (96% vs. 71% for IME_Rho_tet(32), *P*<10^−7^). Finally, other RPP genes were primarily found in bacteria of human and chicken origin, but no statistical test could be performed because of the limited number of genomes (Fig. 4b).

### Taxonomic distribution of gut-associated bacteria carrying IME_Rho_tet and associated RPPs

**Fig. 5.**
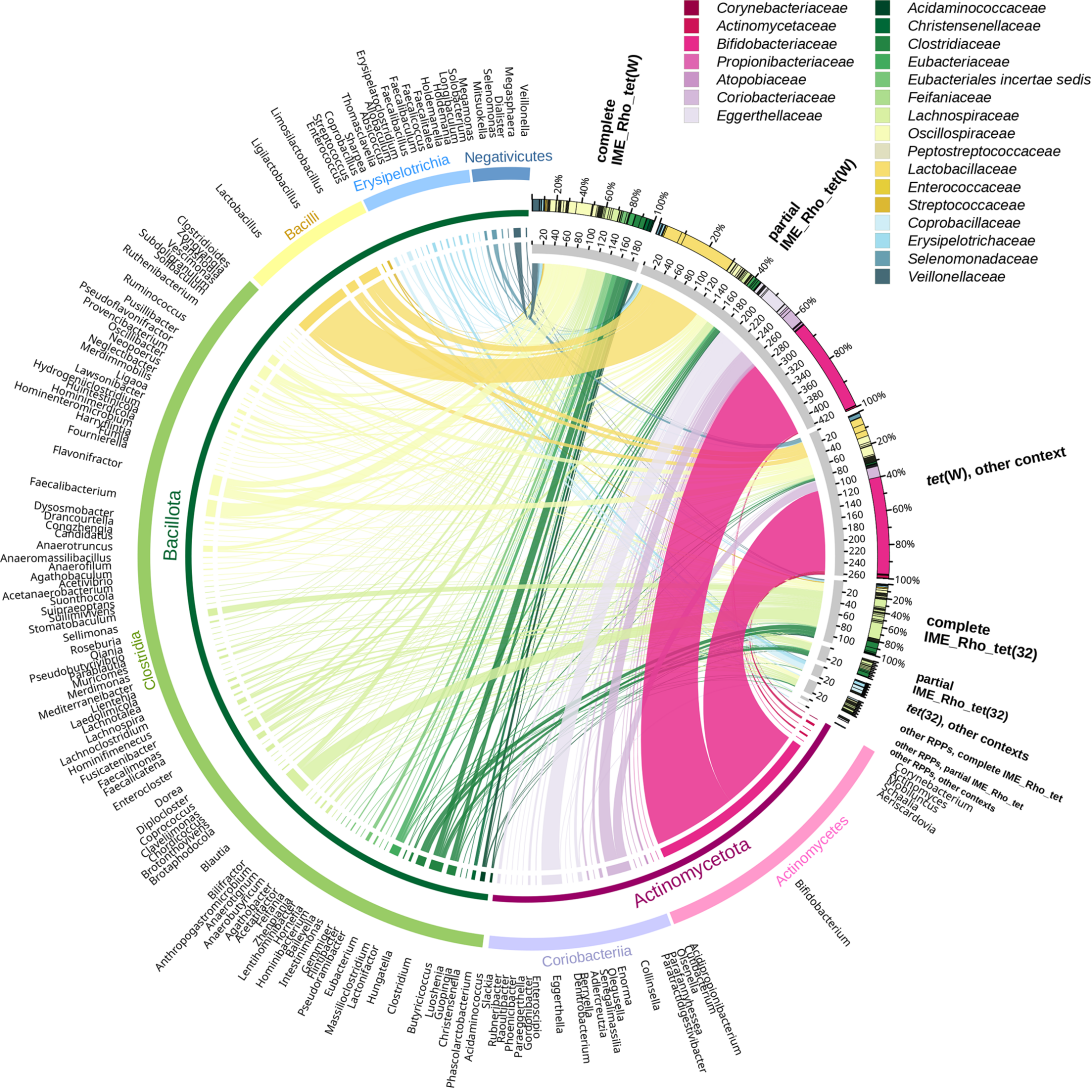
Taxonomy of RPP-carrying gut bacteria, *tet*(O) and *tet*(O/32/O) mosaic genes excluded. Only RPP-carrying genomes for which the host source was known were included. Bacterial genera are indicated on the left and RPP genes are indicated on the right, grouped by gene and genetic context (complete IME_Rho_tet, partial IME_Rho_tet, other genetic contexts). Thick grey lines below each group represent the number of genomes. Bacterial classes and phyla are depicted with thick and thin coloured lines below the genera labels, respectively. Genus-to-RPP links are coloured according to the bacterial family and their width is proportional to the number of genomes from each group. Diagram created using Circos v.69.9 [60].

In our dataset, complete IME_Rho_*tet*(W) were only detected in the *Bacillota* phylum, among 13 families representing 63 genera of gut-associated bacteria. In total, 90% of the elements were associated with members of the *Clostridia* class, mostly from the *Oscillospiraceae* and *Lachnospiraceae* families, but with no clear dominant genera (Fig. 5). Surprisingly, *tet*(W) genes found on partial IME_Rho_tet showed a totally different host bacteria distribution. More than half were distributed among 20 *Actinomycetota* genera, with *Bifidobacterium* species carrying more than 34% of the genes (Fig. 5). The remaining partial IME_Rho_*tet*(W) were found in only 35 *Bacillota* genera, and there was a strong bias towards *Lactobacillaceae*, especially *Lactobacillus* and *Limosilactobacillus*, which carried together 25% of these elements. For *tet*(W) in other contexts, the distribution was even more biased towards *Actinomycetota*, with *Bifidobacterium* making up 57% of the total and *Collinsella* being the second most frequent carrier with 6% of the genes (Fig. 5). Distributions of *tet*(32) and other RPPs associated with complete or partial IME_Rho_tet mirrored complete IME_Rho_tet(W) distribution, with more than 90% of the copies being carried by *Oscillospiraceae*, *Lachnospiraceae* or *Clostridiaceae* and virtually no *Actinomycetota* or *Lactobacillaceae* (Fig. 5). Only *tet*(32) not linked to IME_Rho_tet showed specific a bacterial host pattern, since they were primarily found on *Thomasclavelia* and *Faecalibacillus* species, two members of the *Erysipelotrichia* class only rarely carrying the RPPs studied here.

The analysis of the distribution of the four most represented bacterial families carrying *tet(*W) (*Oscillospiraceae*, *Lachnospiraceae*, *Lactobacillaceae* and *Bifidobacteriaceae*) among host sources indicated that *Bifidobacteriaceae* mostly originated from the human gut, whatever the genomic context (Eagon Pearson test adjusted residuals, *P*<10^−8^ each). On the contrary, *Lactobacillaceae* were significantly more associated with chickens, pigs and other domestic animals when carrying partial IME_Rho_tet(W) (*P*<10^−4^ each) and with pigs and other domestic animals when carrying *tet*(W) not linked to IME_Rho_tet (*P*<10^−8^ and *P*<10^−3^, respectively). Overall, our data indicate that IME_Rho_tet has a wide host range, spreading among *Clostridia* and *Erysipelotrichia* bacterial classes whatever the carried RPP, while *tet*(W) found outside of complete IME_Rho_tet has an even wider host range, including *Actinomycetota* and *Lactobacillaceae* hosts.

### Genetic context of IME_Rho_tet and comparison with other known mobile elements carrying *tet*(*W*) or *tet*(32)

**Fig. 6.**
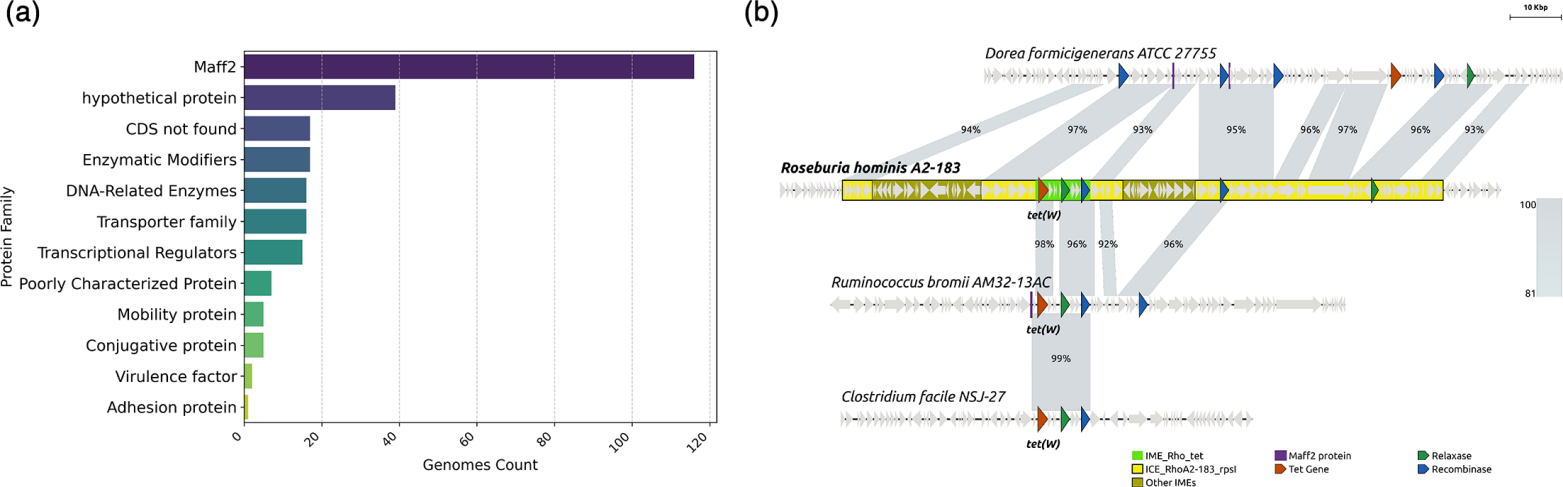
Genetic context of IME_Rho_tet. (a) Proteins interrupted by IME_Rho_tet elements, grouped by functional families. Functional annotations were collected from regions homologous to integration sites of each IME_Rho_tet for which both TIRs could be identified. (b) Comparison of genomic regions of IME_Rho_tet from *R. hominis* A2-183 and other highly similar elements in *Ruminococcus bromii* AM32-13AC (NZ_QSIY01000002) and *Clostridium facile* NSJ-27 (NZ_JACOQK010000001). Manual annotations from FirmiData [31] were used to display MGE regions in *R. hominis* A2-183 (NC_015977). The region in *D. formicigenerans* ATCC 27755 (NZ_CP102279) similar to ICE_RhoA-183_rpsI is also displayed to highlight the precise integration of IME_Rho_tet. ORFs encoding RPPs, relaxases, recombinases/integrases and MAFF2-family proteins are, respectively, displayed in orange, green, blue and purple; other ORFs are displayed in light grey. Picture created using Genofig v.1.1 [57].

Integration sites of the detected IME_Rho_tet were computationally recreated from the proximal 5′- and 3′-150 bp flanking regions and searched in the whole GenBank nucleotide database. Among the 256 IME_Rho_tet for which the recreated integration site matched a sequence in GenBank, 116 (45%) were integrated in small ORFs (130–250 bp) encoding MAFF2-related proteins (Fig. 6a). These proteins of unknown function were named based on their first four amino acids and were first identified as associated with *tet*(W) [45]. In our dataset, these MAFF2 integration sites form 48 clusters at 90% nucleotide similarity with 10–43% divergence between clusters (mean 29%), indicative of a high dissemination potential for IME_Rho_tet. It should also be noted that 36 of the 39 recreated integration sites encoding hypothetical proteins (Fig. 6a) share extensive similarity (72–84%) with MAFF2-encoding ORFs, suggesting that they are actually MAFF2 proteins encoding genes not properly annotated. ORFs encoding MAFF2 were also found adjacent to *tet*(W) in ICE_RbtetW_07 [16], while in the FirmiData-annotated sequence of *R. hominis* A2-183, IME_Rho_tet is located on a putative ICE integrated in the ribosomal protein *rpsI*, named ICE_RhoA2-183_rpslI (Fig. 6b). It is therefore possible that elements related to ICE_RhoA2-183_rpsI, rather than IME_Rho_tet itself, are the dissemination vectors of *tet*(W) and *tet*(32). However, this ICE also exists with more than 93% nucleotide identity but without IME_Rho_tet in *Dorea formicigenerans* ATCC 27755, indicating that the latter is able to excise from and/or insert into the MAFF2-encoding gene (Fig. 6b). Almost identical IME_Rho_tet elements were also found integrated in MAFF2-encoding ORFs of more distant putative MGEs or in other unrelated contexts from different genera (Fig. 6b), further supporting the independent mobility of IME_Rho_tet.

The gene *tet*(W) was previously reported on several MGEs of gut-associated bacteria, such as the transposon Tn*B1230* in *Butyrivibrio fibrisolvens* 1.230 [18] or ICE_RbtetW_07 in *Blautia schinkii* DSM 10518 [16]. When comparing the sequence of these MGEs with those of IME_Rho_tet, it appears that both are actually linked to IME_Rho_tet: ICE_RbtetW_07 is an ICE closely related to ICE_RhoA2-183_rpsI and carries a complete IME_Rho_tet, while Tn*B1230* is a remnant of IME_Rho_tet which endured genetic rearrangement (Fig. S4). Similarly, the genomic island recently identified to carry *tet*(32) in *Streptococcus pneumoniae* 131016 [46] also carries a complete IME_Rho_tet (Fig. S4). Although *tet*(W)-carrying MGEs reported in the literature in *Streptococci* (ICE_LysS, phiSsUD.1, Tn*5252*) do not harbour a complete IME_Rho_tet, they all show strong homology to the beginning of the element, including its 5′ TIR (Fig. S5a). The presence of *tet*(W) in these bacteria from the oral microbiota, therefore, likely originates from former IME_Rho_tet integrations, which degenerated over time. Finally, comparisons with MGEs described in bacteria from the phylum *Actinobacteriota* provide more mitigated results. The whole 5′ region of IME_Rho_tet could be detected in ATE-2 and ATE-3 elements from *T. pyogenes*, while ATE-1 and the plasmid pJA144188 from *Corynebacterium resistens* harbour only *tet*(W) with no other trace of IME_Rho_tet sequence (Fig. S5b).

## Discussion

RPP-encoding genes are the most prevalent ARGs in human and livestock gut microbiota, and among them, *tet*(W) is frequently found as the most abundant. Here, we show that the putative mobile element IME_Rho_tet is a major contributor to *tet*(W) dissemination, with 68% of all detected *tet*(W) associated with complete or partial IME_Rho_tet. Although the MAFF2-encoding ORF into which this MGE is inserted is part of larger mobile elements, we showed that IME_Rho_tet is most likely the genetic unit mobilizing *tet*(W). Beyond *tet*(W), our results show that IME_Rho_tet can mobilize a variety of other RPPs. It is particularly true for *tet*(32), with 88% of the genes being associated with the IME_Rho_tet element. Since this RPP is also among the most abundant resistance genes in gut microbiota [47], it further underscores the importance of IME_Rho_tet in ARG dissemination in this ecosystem. The extensive nucleotide diversity observed at the RPP and relaxase loci of IME_Rho_tet, despite a highly conserved structure, suggests that the element has evolved through homologous recombination, probably fuelled by the fitness advantage provided by the RPP. Although antibiotic pressure usually results in structural changes of MGEs by insertions and rearrangements [48,49], presence of structurally identical but highly divergent regions has already been observed in other mobile elements, notably at the RPP locus among members of the Tn*916* family [50,51].

The mobility of *tet*(W) has long been puzzling because of the various genetic contexts described in the literature. We have shown that most of the previously reported *tet*(W)- and *tet*(32)-carrying mobile elements such as Tn*B1230*, ICE_RbtetW_07, ATE-2 and ATE-3 or MGEs found in oral *Streptococci* actually include complete or partial IME_Rho_tet. Moreover, genetic cassettes proposed to mobilize *tet*(W) in Kazimierczak *et al.* [45] also correspond to the element, since their conserved upstream 657 nucleotides and downstream ORFY are homologous to the region up to the 5′ TIR and to the SAM-dependent methyltransferase of IME_Rho_tet, respectively. It is not the case, however, for ATE-1 and pJA144188, two MGEs described in *Actinomycetota* species. Active dissemination of these two elements or related ones may explain the high proportion of *Actinomycetota* genomes carrying *tet*(W) not linked to IME_Rho_tet in our dataset. Another possibility is that these *tet*(W) genes could be intrinsic, as suggested by a recent study showing that almost all *Bifidobacterium animalis* subsp. *lactis* strains carry a chromosomally encoded variant distinct from other *tet*(W) and located in a conserved genomic context not related to an MGE [52]. Whether some gut-associated *Bifidobacteria* species could be the original source of *tet*(W) is an intriguing hypothesis that would deserve further investigation.

One limitation of our study is that our work was based on sequenced genomes and may not reflect the actual contribution of IME_Rho_tet to the abundance of *tet*(W) in gut microbiota revealed by metagenomic studies. For instance, if *Bifidobacterium* spp. were the main components of gut microbiota, *tet*(W) abundance would be mainly caused by MGEs other than IME_Rho_tet. In the flora of healthy humans, members of the *Bacilli* class (*Lactobacillus* spp., *Enterococcus* spp.) and *Actinomycetota* phylum (e.g. *Bifidobacterium*), which carry most *tet*(W) not associated with complete IME_Rho_tet, are outnumbered by several orders of magnitude by genera from the *Clostridia* class, into which all complete IME_Rho_tet were detected [4]. By contrast, *Lactobacillus* species were reported to be highly abundant in swine gut and chicken small intestine [5,6], consistent with our observation that this genus contributes more than expected to the dissemination of *tet*(W) in these hosts, although not via complete IME_Rho_tet. We therefore anticipate that IME_Rho_tet has a major role in *tet*(W) and *tet*(32) abundance in human gut microbiota, while its influence may be less pronounced in livestock animals. Using hybridization baits to enrich for DNA carrying RPPs identified in this study, followed by long-read sequencing, would help to confirm these hypotheses [53,54].

## Conclusion

Understanding the genetic vectors mobilizing antibiotic resistance determinants is a fundamental step to achieve efficient policy control of antimicrobial resistance emergence. Primary vectors of several RPP genes are well-documented, such as CTnDOT spreading *tet*(Q) in *Bacteroidetes* spp. [55], transposons of the Tn*5252* family spreading *tet*(O) in *Streptococci* [28] and of course Tn*916* and its derivative, representing the main vector of *tet*(M) dissemination in *Bacillota* [56]. Our identification of the IME_Rho_tet family as a major carrier of *tet*(W) and *tet*(32) fills the gaps that remained for these two genes. Nonetheless, our observations are currently *in silico* predictions only, and experimental validation of IME_Rho_tet transfers needs to be carried out. Since IMEs are non-autonomous elements [43], deeper bioinformatic analyses are first required to determine the helper elements triggering IME_Rho_tet excision and conjugative horizontal transfer.

## Supplementary material

10.1099/mgen.0.001640 Table S1.

10.1099/mgen.0.001640 Table S2.

## Abbreviations

ARGs: antibiotic resistance genes
MGEs: mobile genetic elements
RPP: ribosomal protection protein
IME: integrative mobilizable element
TIRs: terminal inverted repeats
